# Extracellular succinate derived from ectopic milieu drives adhesion and implantation growth of ectopic endometrial stromal cells via the SUCNR1 signal in endometriosis

**DOI:** 10.1186/s12964-023-01415-7

**Published:** 2024-01-30

**Authors:** Qi Tian, Jingyao Ruan, Yuning Wang, Yinping Xiao, Qi Cheng, Yun Chen, Mingqing Li, Kaikai Chang, Xiaofang Yi

**Affiliations:** 1 Department of Gynecology, Hospital of Obstetrics and Gynecology, Fudan University, 419# Fangxie Road, Shanghai, 200011 China; 2 Department of Pathology, Hospital of Obstetrics and Gynecology, Fudan University, 419# Fangxie Road, Shanghai, 200011 China; 3 Laboratory for Reproductive Immunology, Hospital of Obstetrics and Gynecology, Fudan University, Shanghai, 200011 People’s Republic of China; 4 Shanghai Key Laboratory of Female Reproductive Endocrine Related Diseases, Shanghai, China

**Keywords:** Endometriosis, Succinate, Peritoneal mesothelial cell, Endometrial stromal cells (ESCs), Macrophage, SUCNR1

## Abstract

**Background:**

As a dual-function metabolite, succinate has emerged in cell function and plays a key signaling role in linking mitochondrial function to other cellular functions. Succinate accumulation in the cytoplasm is commonly associated with hypoxia in the microenvironment and immune cell activation. Extracellular succinate released into the microenvironment is considered an inflammatory alarm that can be sensed by its membrane receptor SUCNR1, which boosts proinflammatory responses and acts akin to classical hormones and cytokines. Succinate plays an important role in the development of inflammatory diseases. Whether succinate facilitates the progression of endometriosis (EMs), characterized by chronic inflammation and peritoneal adhesion, is worth exploring.

**Objective:**

We mimicked the ectopic milieu in vitro and in vivo to evaluate the main source and potential role of succinate in endometriosis. We assessed the molecular and functional effects of succinate on macrophages and peritoneal mesothelial cells in peritoneal cavity. The effect of succinate/SUCNR1 signaling on ectopic endometrial stromal cells (ESCs) was further explored in this study.

**Methods:**

In this study, we used targeted organic acid metabolomics analysis and in vitro assays to assess the potential accumulation of succinate in the peritoneal fluid of EMs patients. We examined its correlation with disease severity, Visual Analogue Scale, and the Endometriosis Fertility Index. Flow cytometry, enzyme linked immunosorbent assay, western blot assay, quantitative real-time PCR, and other molecular biology techniques were used to explore the potential mechanisms.

**Results:**

By mimicking the ectopic milieu, we constructed an in vitro co-culture system and found that M1 polarized macrophages and that the peritoneal mesothelial cell line (HMrSV5) mainly released succinate into their microenvironment and activated the succinate receptor (SUCNR1) signal, which further polarized the macrophages and significantly enhanced the invasive survival of ESCs, and the adhesion to the peritoneum. We further investigated the pathological effects of extracellular succinate in vivo using a xenograft mouse models of endometriosis.

**Conclusions:**

Succinate-SUCNR1 signaling facilitates the creation of inflammatory cells and plays a vital role in EMs progression and peritoneal adhesion. Our work on the molecular mechanisms underlying succinate accumulation and function will help elucidate the phenotypic mysteries of pain and infertility in EMs.

Video Abstract

**Supplementary Information:**

The online version contains supplementary material available at 10.1186/s12964-023-01415-7.

## Introduction

Endometriosis (EMs) is a heterogeneous clinical syndrome characterized by a chronic inflammatory process strongly linked to peritoneal adhesions, infertility, dysmenorrhea, and chronic pelvic pain [1]. The endometrial stromal cells (ESCs) and epithelial cells within the retrograde menstrual endometrium are commonly attached to the pelvic peritoneum. Once ectopic lesion is formed, the ectopic tissue with periodic bleeding is exposed to immune surveillance, leading to chronic inflammation and repeated tissue repair. The presence of cytokines and shifts in circulating immune cell populations create a widespread inflammatory environment and the development of peritoneal adhesions (PA) extending outside the pelvis [2]. Monolayers of peritoneal mesothelial cells and macrophages form major cell populations in the peritoneal fluid, which may play a central role in lesion establishment and maintenance by driving chronic inflammation and tissue remodeling. Although the pathogenesis of EMs remains unclear, genetics and the microenvironment are its key drivers [3].

Succinate occupies an extremely vital position in metabolism because of its direct connection with the Krebs cycle and the mitochondrial respiratory chain [4, 5]. The final stage of the Krebs cycle involves the regeneration of oxaloacetate through a process wherein succinate is oxidited to fumarate via succinate dehydrogenase (SDH) [6]. Dynamic changes in SDH under physiological or pathological metabolic conditions are associated with succinate accumulation [7, 8]. Over the past 10 years, new roles for extracellular succinate have expanded beyond metabolism and signaling. Beyond its metabolic role in conditions of stress and damage, an increasing body of evidence points to additional immunological functions, especially in subacute inflammatory conditions such as inflammatory bowel disease, Crohn’s disease [9, 10], gestational diabetes [11], and non-alcoholic fatty liver disease [12]. Succinate accumulation is followed by succinate release from cells, which then acts on other cell types via SUCNR1, driving inflammation or type 2 immunity [5]. Recently, it is interesting to find that succinate can either by synergistically activated with estrogen [13],or through downregulating voltage-gated potassium channel subfamily Q member 1 (KCNQ1) levels to promote the growth of endometrial cancer cells in vitro and in vivo [14]. Decreased succinate accumulation contributes toward the onset of abortion in mice [7]. These multiple functions indicate that succinate plays important roles in cellular activation. Succinate triggers macrophage polarization and subsequent inflammation. However, the role of succinate-SUCNR1 signaling in EMs, which is characterized by chronic inflammation, remains unclear.

Hence, we hypothesized that stimulation with cytokines, such as IL-6, or contact with endometrial stromal cells during retrograde menstruation triggers succinate release from peritoneal mesothelial cells (PMCs) and macrophages. Extracellular succinate polarizes macrophages into the M1-like type and continuously recruits them via CCL2 secretion from PMCs. Thus, crosstalk between these cells leads to massive succinate accumulation and an inflammatory microenvironment. Eventually, the accumulated succinate enhances the survival, adhesion and deep infiltration of ESCs via SUCNR1 signaling, leading to the acceleration of EMs progression.

## Materials and methods

### Patients and tissues collection

Premenopausal women diagnosed with EMs (endometrioma, peritoneal endometriosis, deep infiltrating endometriosis) or other benign gynecological diseases underwent laparoscopic surgery at the Obstetrics and Gynecology Hospital of Fudan University between January 2020 and December 2022. Normal endometrial samples were obtained from six patients without EMs who underwent combined laparoscopy and hysteroscopy for tubal infertility and uterine septum. EMs was diagnosed based on clinical symptoms and imaging findings. Symptoms related to EMs include pelvic masses, chronic pelvic pain, dysmenorrhea, dyspareunia, infertility, and cyclical alterations in bowel and urinary habits that occur only during menstruation. A total of 2–10 ml of undiluted peritoneal fluid was drawn at the beginning of the laparoscopy, and biopsies from ectopic lesions were obtained from each patient. Finally, according to the revised American Fertility Society (rAFS) (ASRM, 1997), patients with pathologically confirmed EMs were grouped according to the disease stage (stage I-II, *n* = 12; stage III-IV, *n* = 24). Thirty patients without EMs were included in the control group, and peritoneal fluid was collected during laparoscopic surgery. Histopathological examination post-surgery confirmed the diagnosis of benign gynaecological diseases, including corpus luteum cyst (*n* = 3), teratomas (*n* = 8), fibroids (*n* = 10), mesosalpinx cyst (*n* = 4), female genital anomalies (*n* = 3), infertility without PID (*n* = 2). We excluded patients with pelvic inflammatory disease or secondary infertility with pelvic inflammatory disease when collecting samples from control or endometriosis group.

The enrolled patients were free of hormonal medications for at least 6 months. Patients with acute or subacute inflammatory diseases, autoimmune disorders, pregnancy, or malignant tumors were excluded from the study. The clinical and demographic characteristics of all participants are shown in Supplementary Table 1.

### Peritoneal fluid isolation

Peritoneal fluid was promptly cooled on ice upon collection and then transferred to the laboratory within 30 min for further experiments. Fresh peritoneal fluid was centrifuged at 1500 rpm(4 °C) for 5 min. The pellet was resuspended, and an erythrocyte lysis solution (1×) was added according to the manufacturer’s instructions. After centrifugation (1000 rpm) three times, fresh cell pellets were immediately prepared for flow cytometry. The supernatant of theperitoneal fluid cells and debris was packaged in 1.5-mL centrifugal tubes and stored in a refrigerator at − 80 °C until metabolomics detection and enzyme-linked immunosorbent assay (ELISA) analysis.

### Targeting organic acid metabolomics analysis

Shanghai Lu-Ming Biotech Company Limited (Shanghai, China) provided an experimental platform and assistance for the target organic acid metabolomics analysis. Briefly, a mixture of acetonitrile and methanol (2:1, v/v, containing seven isotopes internal standards) was used to collect 0.1 ml per sample. After shaking and centrifugation, 100 μl of supernatant per sample was freeze-dried. Finally, a mixture of BSTFA and n-hexane (4:1, v/v) was added to the sample, vortexed vigorously for 2 min, and derivatized at 70 °C for 60 min. The samples were analyzed using a gas chromatography system Trace1310 coupled with a TSQ9000 Mass spectrometer equipped with an electron ionization (EI) source (Thermo Fisher Scientific, USA).

The raw data exported by UPLC-MS/MS were processed using the QuanMET software (v1.0, Metabo-Profile, Shanghai, China). The concentrations and peak areas of the standards were used to construct a standard curve and calculate the sample concentration. The calculated concentrations of bile acids in all samples were imported into SIMCA-P+ software (v. 14.1, Umetrics, Sweden) for multivariate analysis, including principal component analysis and orthogonal partial least squares-discriminant analysis (OPLS-DA). An independent sample non-parametric test was used to assess significant differences between the groups (*P* < 0.05), and the variable of importance values in the OPLS-DA model were used to identify potential biomarkers.

### Cell culture and treatments

Primary human endometrial stromal cells (hESCs) from the endometrium of patients with and without EMs were isolated using collagenase digestion, as previously described [15], and cultured in Dulbecco’s Modified Eagle Medium/Nutrient Mixture F-12 (DMEM/F12) supplemented with 10% fetal bovine serum (FBS) for flow cytometry (FCM) analysis.

The cell line used in this study, human endometrial stromal cells (hESCs) in our laboratory for Reproductive Immunology were obtained from the American Type Culture Collection (CRL-4003; ATCC, Manassas, VA, USA),. hESCs were cultured in DMEM/F12(10% FBS) for the co-culture system and other assays.

HMrSV5 and THP-1 cells were procured from the National Collection of Authenticated Cell Cultures of China. HMrSV5 cells cultured in DMEM F12(10% FBS) and THP-1 cells cultured in RPMI-1640 (10% FBS) were incubated with or without various concentrations of drugs for predefined times before each experiment, according to the cell experimental protocol.

For macrophage polarization, THP-1 cells were differentiated and polarized by using 100 ng/ml phorbol 12-myristate13-acetate (PMA; Sigma-Aldrich) for 48 h to obtain M0, and M0 were transformed into M1 through lipopolysaccharide (LPS) (Peprotech) (100 ng/ml) and IFN-γ (PeproTech)(20 ng/ml) stimulation or M2 through IL-4 (PeproTech) (20 ng/ml) and IL-13 (PeproTech) (20 ng/ml) stimulation for 48 h. FCM was conducted to verify successful induction via CD80, CD86, CD163, and CD206. Non-adherent macrophages were washed with phosphate-buffered saline (PBS), and adherent cells were cultured in fresh RPMI-1640 medium.

The details of cytokines used in this study are as follows: IFN-γ Peprotech Cat#300–02 lot#091927; LPS Peprotech Cat#M9524; IL-4 Peprotech Cat#200–04 lot#051914; IL-10 Peprotech Cat#200–10 lot#11021; IL-6 Peprotech Cat#200–06; IL-13 Peprotech Cat#200–13 lot#102123.

### Cell viability assays

Cell viability was measured using the cell counting kit-8(CCK-8). hESCs or HMrSV5 cells (four replicates per group) were seeded in 96-well plates (Corning) with 100 ml medium (10% FBS) and then incubated at 37 °C overnight. Following a 48-hour stimulation with succinate, cell supernatants were removed. Subsequently, CCK-8 solution (10 μl) and culture medium (100 μl) were added into each well. After incubation for another 1 h at 37 °C, the plates were measured via a microplate reader at an absorbance of 450 nm (Bio-Rad 680, Bio-Rad, USA).

### Apoptosis assays

For the apoptosis assay, hESCs or HMrSV5 cells were seeded in 24-well plates and cultured with LPS or succinate for 48 h. Cells were then co-stained with Annexin V-PE (BD Pharmingen, Heidelberg, Germany) and 7AAD. Flow cytometry (Beckman) was performed to obtain data on apoptosis, which were analyzed using FlowJo software.

### Scratch wound assay

The hESCs (1 × 10^5^ cells/well, four replicates per group) were seeded in a 12-well plate. After reaching confluence, cells were scratched using a sterile tip to mimic the shape of the wound. FBS-free medium was used to wash and remove loose cells. hESCs were then treated with succinate (0, 1, 2.5, or 5 mM) and photographed at 0, 24, and 48 h by using a light microscope. The closure area of wound was calculated as follows: Wound Closure (%) = ((Primary wound size-Final wound size)*100%/ Primary wound size.

### Matrigel invasion, chemotaxis and adhesion assays

In the transwell assay, hESCs or HMrSV5 (1 × 10^4^ cells/well, three replicates per group) were seeded into the upper chamber of 24-well transwell plates (8 μm pore filters) (Corning, USA). The lower chamber was supplemented with medium (10% FBS) containing either succinate (0, 1, 2.5, 5 mM) or CCL-2 (Abclone Cat#RP01411) (100 ng/ml). After 48 h, migrated cells on the lower surface were stained and observed under a microscope.

For chemotaxis assay, hESCs (1 × 10^4^ cells/well, three replicates per group) were seeded in the upper chamber of 24-well transwell plates (8 μm pore filters) (Corning, USA), and HMrSV5 cells (1 × 10^4^ cells/well) stimulated with succinate (0, 2.5 mM) or CCL2 (100 ng/ml) were paced in the lower chamber. CCL2 treatment was used as a positive control. After 48 h, hESCs were replaced with THP-1 cells, which were pre-labeled with CellTracker red (DiO, Beyotime, C1995S, China), and then continuously incubated for another 12 h with 3 μm pore filters. Finally, the traced THP-1 cells were collected and calculated as cell number per field (scale bar-100 μm) (red tracer staining THP-1 cell).

For the adhesion assay, HMrSV5 cells (2 × 10^5^ cells/well; three replicates per group) were treated with succinate (0, 1, 2.5, 5 mM) and seeded in 6-well plates. hESCs(1 × 10^4^ cells/well; three replicates per group) pre-labeled with CellTracker green (DiO, Beyotime, C1993S, China) were seeded into each HMrSV5-well. Finally, the traced hESCs were calculated as cell number per field using a fluorescence microscope (scale bar-200 μm) (green tracer staining hESCs).

### Flow cytometry

Peritoneal cell pellets collected from the peritoneal fluid were suspended in PBS and stained with the following antibodies: anti-human CD14 -PerCy5.5 (BioLegend,325,621), anti-human CD45 APC-Cy7 (BioLegend, 368,515) and anti-human GPR91/SUCNR1 FITC (Alomone, ASR-090-F). After staining for half an hour, the cells were washed and prepared for FCM (Beckman Coulter). Data were analyzed using FlowJo (v10) software.

Flow cytometry was also performed to analyze the expression of CD80, CD86, CD163, CD206 and MCT1 in macrophages in vivo or THP-1 cells in vitro, as well as SUCNR1 levels in hESCs. The FCM antibodies used were as follows: anti-human CD86 Percp/cy5.5 (BioLegend, 305,419); anti-human/mouse MCT1 PE (R&D, FAB8275P); anti-human/Mouse GPR91/SUCNR1 FITC (Alomone, ASR-090-F); anti-mouse CD45 percp (BioLegend, 103,129); anti-human/mouse GPR91/SUCNR1 FITC (Alomone, ASR-090-F); anti-mouse CD80 PE (BioLegend, 104,707), anti-mouse CD86 ALexa fluor 700 (BioLegend, 105,024); anti-mouse CD163 BV421 (BioLegend, 155,309); anti-mouse CD206 BV605 (BioLegend, 141,721), anti-mouse CD11b PCy 7(BioLegend, 101,215); anti-mouse F4/80 APC (BioLegend, 123,116).

### ELISA assay

The levels of succinate in the peritoneal fluid and cell supernatants were measured using ELISA. Briefly, HMrSV5 cells, hESCs, and macrophages polarized from THP-1 cells were seeded in 6-well plates. After culturing for 48 h, the cells were treated with BMDM alone, IL-6 (100 ng/ml), or CCL2 (50 ng/ml) for another 48 h. The cell supernatant was centrifuged at 1000 rpm (4 °C) for 10 min. The peritoneal fluid was collected as described above. It was placed on ice upon collection and centrifuged twice at 1500 rpm for 5 minutes at 4 °C to remove the cells. All the samples were diluted twice and analyzed according to the specifications of the ELISA kit (Abcam, ab204718).

### Quantitative real-time PCR (RT-qPCR) analysis

Total RNA was extracted via RNA Purification Kit (EZBioscience, USA) and reverse-transcribed into cDNA by using Hifair®II 1st Strand cDNA Synthesis SuperMix for qPCR (Yeasen, Shanghai, China). The quantitative real-time PCR (RT-qPCR) was performed according to the manufacturer’s protocol (Hieff UNICON Universal Blue qPCR SYBR Green Master Mix,Yeasen). Data analysis was repeated three times and analyzed using 2^−ΔΔ^Ct method. The primer sequences used in this study are listed in Supplementary Table 2.

### Immunohistochemistry (IHC)

Tissue microarrays (TMAs) [16] were previously constructed with endometriosis lesions tissues. Immunohistochemistry (IHC) for succinate dehydrogenase complex iron sulfur subunit (SDHB) was performed according to the manufacturer’s instructions. Briefly, after dewaxing and antigen repair, the primary antibody against SDHB (Abcam, ab178423) was incubated overnight(4 °C) at a 1:150 dilution. Subsequently, the membranes were incubated with secondary antibody at room temperature (24–26 °C) for half an hour. Sections were stained with hematoxylin and photographed under a microscope.

### Western blotting assay

HMrSV5 cells (2 × 10^5^ cells/well) were seeded in 6-well plates and treated with different concentrations of succinate (0, 0.5, 1, 2.5, and 5 mM) for 48 h. hESC cells, primary ESC, and primary ectopic ESCs (2 × 10^5^ cells/well) wererespectively seeded in 6-well plates for 48 h. Proteins were extracted by cell lysis. The protein sample (15 μg/lane) was evaluated through electrophoresis and transfected to a 0.45 μm polyvinylidene fluoride (PVDF) membrane, which was stained with enhanced chemiluminescence reagents after incubating with the primary antibody anti-ICAM1 (Abcam, ab53013) (1:1000) or anti-MCT1 (Santa Cruz, sc-365,501) at 4 °C overnight and with a secondary antibody (1:5000) at 24 °C for 2 h. The total gray scale of each strip was quantified using ImageJ software with the values normalized based on housekeeping proteins (i.e., *β*-actin).

### Mouse model of EMs

Thirty adult C57BL/6 female mice (6–8 weeks, weight 20 ± 2 g) were purchased from the Laboratory Animal Facility of Fudan University and used for this study. Animal protocols were approved by the Ethics Committee of the Obstetrics and Gynecology Hospital of Fudan University. All mice were randomly assigned to one of the three groups. Intraperitoneal EMs-like lesions were surgically induced by injecting fragments of the uterine tissue into the peritoneal cavity. Each postoperative mouse received 17-β-Estradiol-3-benzoate (30 *μ* g/kg, Sigma) every 3 days for 14 days. Three days after surgery, each mouse in the experimental group received succinate (100 mg/kg, Sigma) intraperitoneally every 3 days for 14 days. PBS was used instead of succinate in the sham group. In the control group, no surgery was performed and PBS was used instead of succinate. Fourteen days after the operation, endometrial-like lesions were established, the mice were sacrificed, and the peritoneal lavage fluids and ectopic lesions were harvested. SUCNR1 and M1/M2 macrophage markers were measured and analyzed via FCM. MMP9 and ICAM-1 levels in the lesions were detected through IHC.

## Results

### Succinate accumulation in peritoneal fluid and clinical relevance in EMs

**Fig. 1:**
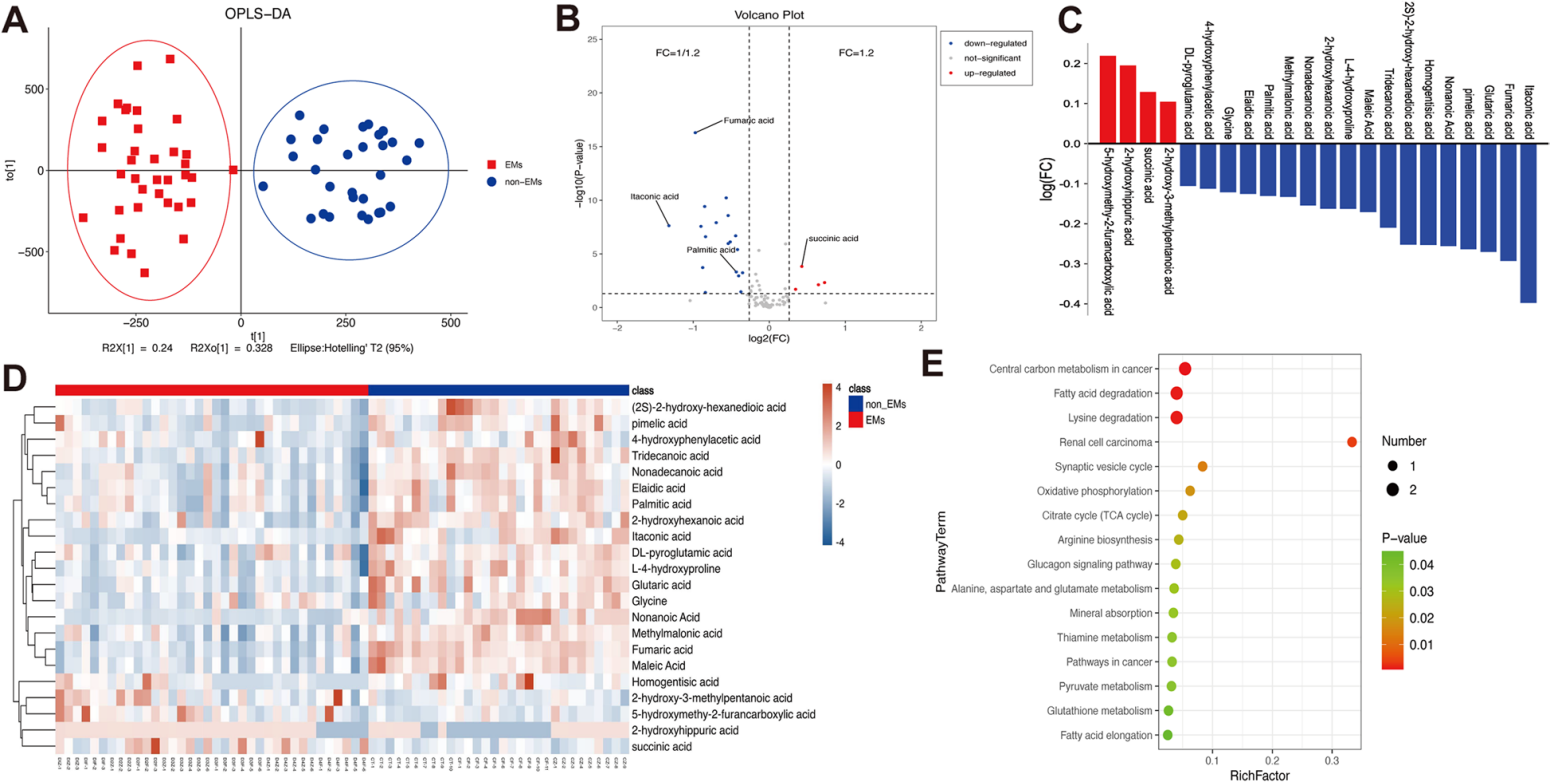
Altered metabolic profiles of peritoneal fluid in EMs compared with Healthy. **A** A PLS-DA score plot of EMs and Non-EMs in cohort. R2X = 0.24, R2Y = 0.231, Q2 = 0.541. **B**, **C** Volcano plot and bar graph of the differential metabolites in EMs and Non-EMs filtered by univariate analysis. **D** Hierarchical clustering of the 22 differential organic acid metabolites (FC > 1.2, *p* < 0.05) in non-EMs and EMs group. Blue indicates a decreased level; red indicates an increased level. **E** Bubble plot for quantitative enrichment analysis showing the metabolic pathway changes between EMs and non-EMs metabolomes in cohort

To assess the levels of metabolites in the peritoneal fluid, we applied targeted organic acid metabolomic analysis to study the differences in organic acid profiles between healthy individuals and EMs patients. The results showed a significantly difference in the peritoneal fluid between the EMs and non-EMs groups (Fig. 1A). Based on the metabolomics results of the peritoneal fluid, four organic acid metabolites, including 5-hydroxymethy-2-furancarbosylic acid, 2-hydroxyhippuric acid, succinic acid, and 2-hydroxy-3-methylpentanoic acid, were clearly elevated in EMs patients (Fig. 1B-D). Specifically, succinate was significantly increased in EMs patients (328.65 ± 105.4 ng/ml) than in those without EMs (244.27 ± 43.76 ng/ml), this finding corresponded with that of the ELISA test **(**Supplementary Fig. 1A). SDHB, a classic mitochondrial enzyme, possesses the unique characteristic of oxidizing succinate to fumarate. Immunohistochemical staining revealed decreased expression of SDHB in ectopic lesions compared to that in the normal endometrium (Supplementary Fig. 1B). Consistent with the result of IHC, the mRNA and protein level of SDHB in ectopic ESC was also lower than than in normal ESCs (Supplementary Fig. 1C,D). Metabolic pathway enrichment analysis showed that pathways were differentially regulated between EMs and non-EMs groups, and the oxidative phosphorylation and citric cycle (Krebs cycle) pathways were significantly upregulated in the EMs group **(**Fig. 1E).

**Fig. 2:**
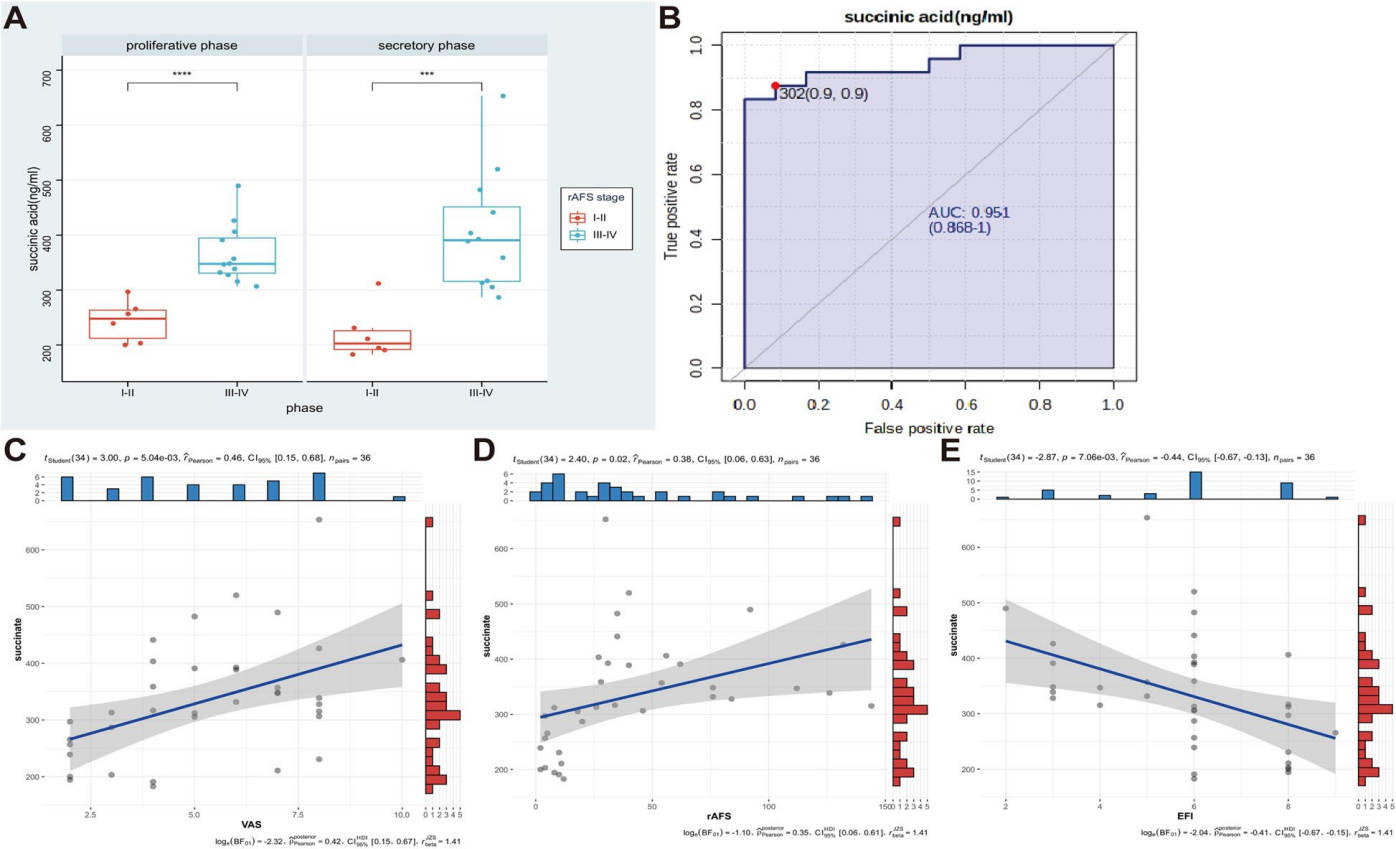
Succinate accumulation of peritoneal fluid from EMs patients is associated with clinical relevance. **A** Level of succinate in peritoneal fluid from EMs patients with different stages. Succinate was assessed via targeting organic acid metabolomics analysis (EMs I-II stage, *n* = 12; III-IV stage, *n* = 24) (Student’s *t*-test). **B** ROC curve for the assessment of EMs severity probability in cohort. **C-E** The Pearson correlation coefficient was used for clinical correlation, and result showed the level of succinate was positively correlated with pain grade VAS (R2 = 0.0.46, *P* < 0.001, 95% confidence interval is 0.15–0.68) and rAFS stage (R2 = 0.38, *P* < 0.02, 95% confidence interval is 0.06–0.63), negative with EFI. ***P < 0.001. VAS: Visual Analogue Scale; rAFS: revised American Fertility Society Scoring; EFI: endometriosis fertility index

Succinate is an important intermediate in intracellular metabolism. Although normally regarded as an intermediate, succinate accumulates under certain pathophysiological conditions, especially at the sites of inflammation and metabolic stress [4, 5, 9, 17–19]. Succinate is not simply an inert byproduct of metabolism but also plays an active role in downstream cellular responses and can have tissue-specific and systemic effects as a proinflammatory mediator [4, 5, 20]. Similarly, we found that higher levels of succinate accumulated in the peritoneal fluid of patients with severe EMs (stage III-IV) than in those with mild EMs (stage I-II) (Fig. 2A). Therefore, we speculated that succinate levels reflect disease severity. Succinate, to some extent, had potential clinical value in reflecting EMs severity (AUC = 0.951) (Fig. 2B). Furthermore, Pearson’s correlation analysis was conducted on the EMs clinical data, and the results revealed a linear correlation between succinate and clinical symptoms/indicators, such as pain (Visual Analogue Scale, R2 = 0.46, *P* < 0.001, 95% confidence interval: 0.15–0.68), EMs stage according to the rAFS score (rAFS,1985) (rAFS, R2 = 0.38, *P* = 0.02, 95% confidence interval: 0.06–0.63), and fertility prediction after EMs surgical staging (Endometriosis Fertility Index [EFI], R2 = − 0.44, *P* < 0.01, 95% confidence interval: − 0.67–-0.13) (Fig. 2C-E). Supplementary Table 1 presents the clinical parameters of the patients with and without EMs.

### Succinate is prone to polarize M1-like macrophages in the endometriotic milieu

Since succinate triggers inflammatory changes in macrophages, changes in gene expression triggered by succinate in M0 macrophages derived from THP-1 cells were compared with the marker gene expression observed in human M1 and M2 macrophages. We conducted RT-qPCR analysis on macrophages exposed to varying concentrations of succinate (0, 0.5, 1, 2.5, and 5 mM) for 48 h. We compared the gene expression changes in M1 markers (CD80, CD86) with M2 markers (CD206, CD163) triggered in M0 macrophages by succinate. As illustrated in Supplementary Fig. 2A-E, in resting M0 macrophages without LPS stimulation, exposure to succinate resulted in the upregulation of genes preferentially expressed by M1 macrophages and the downregulation of genes preferentially expressed in M2 macrophages. Thus, succinate exposure causes the polarization of naïve macrophages toward M1 cells. As previously reported [20, 21], the effects of extracellular succinate and LPS can be superimposed to augment the LPS-driven M1 phenotype **(**Supplementary Fig. 2C). Additionally, we found that succinate markedly induced the transcription of proinflammatory cytokines IL-8, IL-1β, and IL-6 in macrophages; however, a higher concentration of succinate (5 mM) created an opposite effect in IL-8 and IL-6 (Supplementary Fig. 2F-I).

To evaluate the expression of SUCNR1 in M0 macrophages exposed to succinate, we performed the transcription of SUCNR1 via real-time PCR. The results showed dose-dependent expression of *SUCNR1* mRNA in M0 macrophages exposed to succinate as compared to that in the vehicle (Supplementary Fig. 2 J). Succinate appears to activate inflammatory pathways and switch cells to the M1-like phenotype, at least in part, via SUCNR1. These data demonstrate that exposing monocyte-derived macrophages to relevant concentrations of extracellular succinate unequivocally regulates the expression of immune function genes, resulting in the polarization of the M1-like phenotype or synergism with LPS.

### Extracellular succinate is mainly released from M1-polarized macrophages and peritoneal mesothelial cells

**Fig. 3:**
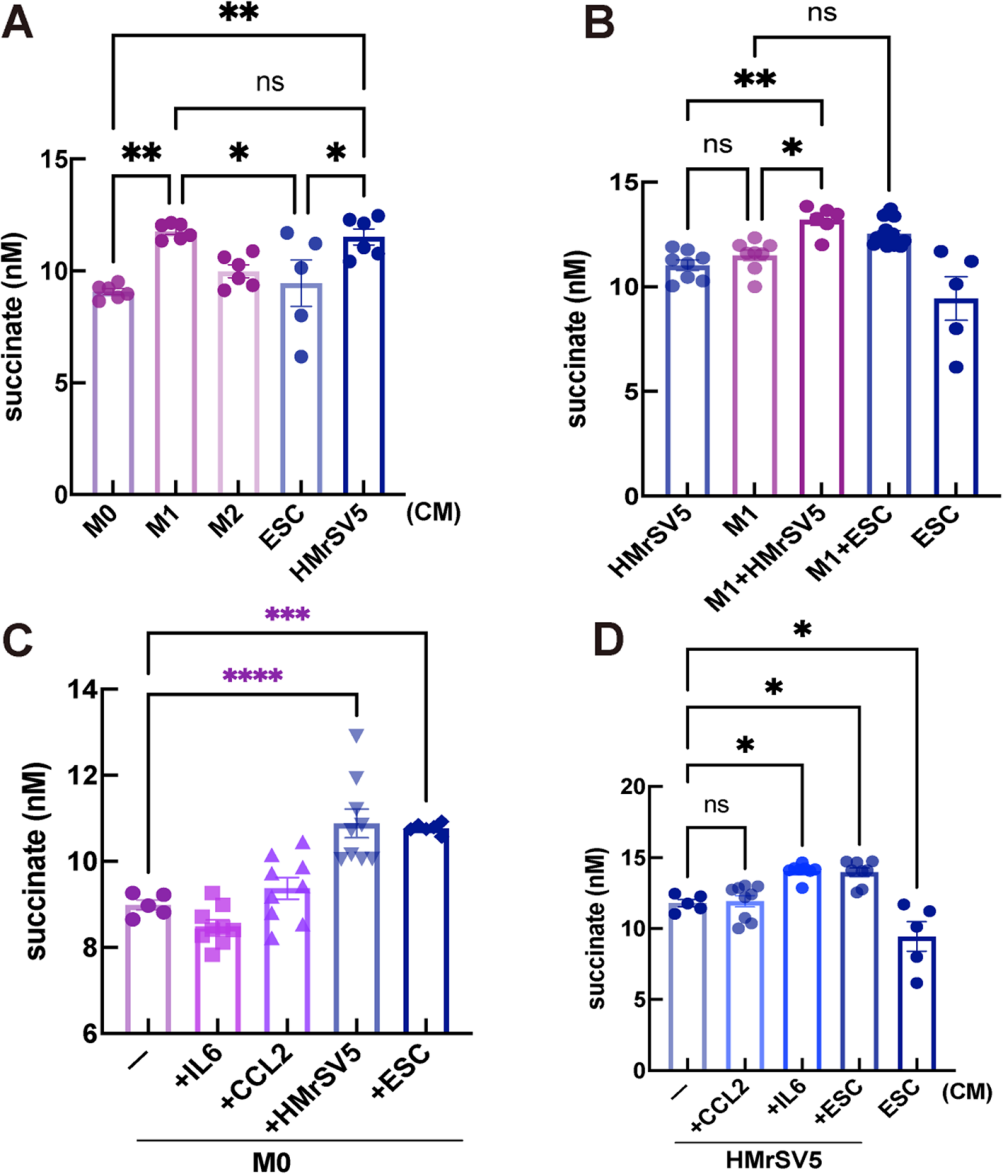
Peritoneal mesothelial cells and M1-polarized Macrophage Mainly Produced Extracellular Succinate. **A** Extracellular succinate secretion measured in different cell types, such as macrophage, HMrSV5 cells and hESCs. **B** The highest level of succinate was in the co-cultured medium of M1-polarized macrophages after co-culture with HMrSV5 cells or hESCs. **C** Naive macrophage state (M0) co-cultured with HMrSV5 cells or hESCs could trigger high level of succinate secretion in the cell medium, but not with IL-6 or CCL2 stimulation. **D** Similarly, extracellular succinate secretion was increased under IL-6 exposure or in co-culture of M1 macrophages and HMrSV5 cells. Statistical significance was assessed either with t test or *one-way ANOVA* followed by *Tukey’s or Holm-Sidak’s post-test*. *P < 0.05, **P < 0.01, ***P < 0.001

Macrophages are the most common population (approximately 60%) of the peritoneal fluid leukocytes in EMs patients. The PMC monolayer that lines the abdominal cavity is the first barrier encountered by menstrual fragments. To understand the capacity of extracellular succinate secretion among these cell types, THP-1 cells were respectively polarized into the M1 phenotype with LPS and IFNγ, or into the M2 phenotype with IL-4 and IL-13 as previously described [22, 23]. Macrophages in different polarizing states were cultured for 48 h, and the levels of extracellular succinate in the cell supernatant were measured using ELISA. Consistently with previous reports that M1 polarized macrophages are the major producers of succinate, our study showed that succinate production in M1 polarized macrophages and PMCs was higher than that in naïve and M2-polarized macrophages (Fig. 3A).

To identify the main source of succinate in the endometriotic milieu, a co-culture model with hESC, PMC line HMrSV5, or THP-1 cells was constructed to imitate the ectopic immune microenvironment of EMs. High levels of succinate were observed in the culture supernatants of M1-polarized macrophages after co-culture with HMrSV5 cells or hESCs (Fig. 3B). As reported in our previous studies, some inflammatory cytokines (such as IL-6 and CCL-2) are significantly elevated in the peritoneal fluid of patients with EMs [15]. Interestingly, the co-culture of resting M0 cells with HMrSV5 cells or hESCs triggered succinate release in macrophages but not when stimulated with IL-6 or CCL2 (Fig. 3C), indicating that contact with hESCs or HMrSV5 cells, rather than the M0 phenotype, determines succinate secretion. In parallel, MCP-1 from the endometriotic milieu failed to induce succinate secretion in HMrSV5 cells. However, IL-6 stimulation or interaction with hESCs significantly increased succinate production (Fig. 3D). Compared with primary normal ESC, ectopic ESC has higher expression of MCT1 and SLC26A6, indicating a stronger ability of succinate exportion (Supplementary Fig. 3A-C). When mimiking ectopic milieu with hESC line and PMCs, channel proteins for intaking succinate elevated in M1 cells, facilitating cell in stress or adaption (Supplementary Fig. 3D-H). Based on these findings, changes in the ectopic milieu establish a vicious circle, with each condition promoting the other and accelerating succinate accumulation. Based on these findings, changes in the ectopic milieu establish a vicious circle, with each condition promoting the other and accelerating succinate accumulation.

### SUCNR1 is elevated in ectopic ESCs and macrophages in EMs patients

**Fig. 4:**
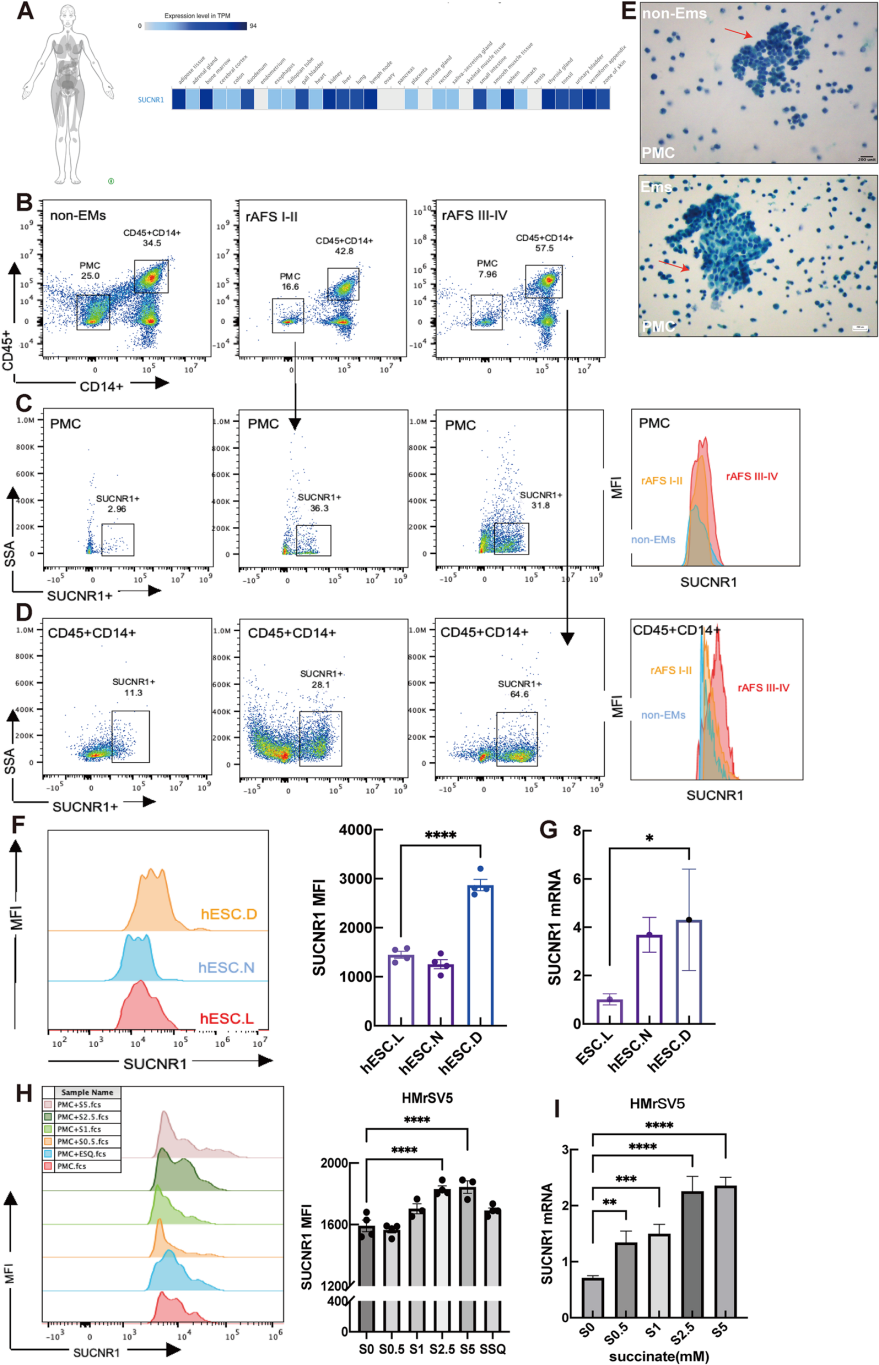
The expression of SUCNR1 in cells from Ectopic milieu. **A** SUCNR1 expression in female tissues from healthy human tissues (data are available from https://www.proteinatlas.org/). **B-D** The level of SUCNR1^+^ macrophages and peritoneal mesothelial cells (PMC) in peritoneal fluid from EMs patients was elevated and correlated with EMs stage. **E** The staining macroscopic observation of peritoneal mesothelial cells (PMC) in peritoneal fluid or intraperitoneal lavage (modified papanicolaou staining, 40X). **F-G** FCM-based MFI assay and qT-PCR analysis designed to measure SUCNR1 expressions, and result showed primary ectopic stromal cell (hESC.D) expressed higher SUCNR1, compared with that of primary normal stromal cell (hESC.N) and human stromal cell line (hESC.N). **H-I** FCM and qT-PCR assays showed the SUCNR1 expression of primary normal ESC stimulated with succiante with different concentrations. The optimal concentration of succinate is 2.5 mM. Significance was assessed either with t test or *one-way ANOVA* followed by *Tukey’s or Holm-Sidak’s post-test.* *p < 0.05, **p < 0.01, ***p < 0.001

To analyze the expression and distribution of SUCNR1 in human tissues, we found, via the Human Protein Atlas/Dataset (data available from proteinatlas.org), that SUCNR1 expression in normal female tissues was low under physiological homeostasis but high in their immune system and gastrointestinal tract (Fig. 4A).

To analyze whether succinate accumulation participates in EMs progression, we measured SUCNR1 expression in CD45^+^CD14^+^ macrophages, PMCs, and primary ESCs from patients with or without EMs via FCM. Serosal healing involves free-floating mesothelial cells [24, 25], and our results showed that macroscopic shed mesothelial cells float in clumps in the peritoneal fluid (Fig. 4E), wiht a 16-fold increase in SUCNR1 expression in the mesothelial cell mass of EMs patients (Fig. 4B,C). Macrophages derived from the peritoneal fluid of EMs patients underwent significant phenotypic changes, demonstrating a 1.8 to 3.9-fold increase in SUCNR1 expression in macrophages (Fig. 4B, D), Additionally, there was a parallel 2-fold increase in SUCNR1 expression in ectopic ESCs (hESC.D) compared with hESC cell line (hESC.L) and primary normal ESCs (hESC.N) (Fig. 4F,G). Therefore, we evaluated the effect of succinate on SUCNR1 expression in HMrSV5 cells. Increasing succinate concentrations induced a dose-dependent increase in SUCNR1 expression in HMrSV5 cells (Fig. 4H,I), indicating the important role of succinate in mesothelial cell function during EMs lesion formation. Using this assay,an optimal concentration of 2.5 mM was determined.

### Succinate enhances ESCs survival and implantation capacity via the SUCNR1

**Fig. 5:**
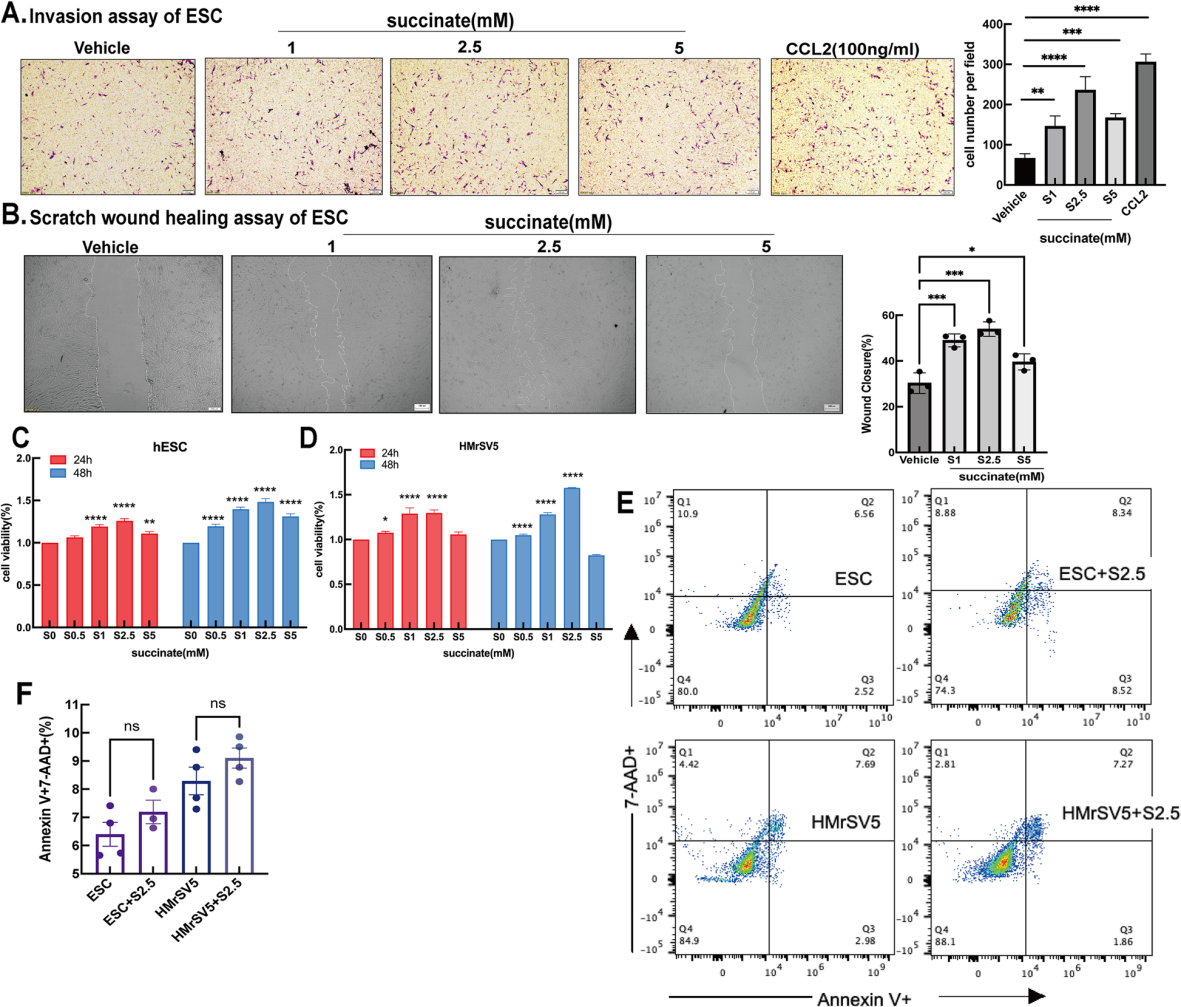
Succinate induces the invasion, wound healing and survival of ESCs. **A** Invasion assays of hESCs. Invasion assay was measured after exposure of hESC to different concentrations of succinate for 48 h, and CCL2 stimulation was used as the positive control (scale bar-200um)(*n* = 3). *One-way ANOV*A, *Dunnett’s* post hoc *test*, *P < 0.05; **P < 0.01; ***P < 0.001. **B** Scrath assays of hESC Scrath assay was conducted after PRE treatment in hESCs with different concentration of succinate for 48 h. Results are from 3 independent trials (*n* ≥ 3 for mimic) and data depicted as column mean graphs with error bars showing confidence intervals (scale bar-200um) *One-way ANOV*A, *Dunnett’s* post hoc *test*, *P < 0.05; **P < 0.01; ***P < 0.001. **C-D** Cell viability of hESCs and HMrSV5 cells was determined by CCK8 assay hESCs or HMrSV5 cells were respectively incubated with different concentrations of succinate for 24 or 48 hours. and then cell viability was determined by CCK8 assay. Data represent mean ± SEM (*n* = 3). *P < 0.05; **P < 0.01; ***P < 0.001. **E-F** Succinate does not affect the apoptosis of hESCs and HMrSV5 cells. hESCs or HMrSV5 cells were cultureed with different concentration of succinate for 48 h, and the proportion of 7AAD^+^ and Annexin V^+^ via FCM was shown in HMrSV5 cells or hESC cells via FCM(*n* = 3). *One-way ANOVA*, *Dunnett’s* post hoc *test*, *P < 0.05; **P < 0.01; ***P < 0.001

Given the importance of extracellular succinate as an immunometabolic signal in the ectopic milieu, we investigated whether succinate released by type 1 proinflammatory macrophages could regulate the activity of the surrounding PMCs in the peritoneal cavity, including that of refluxed and colonized ESCs. Since endometriotic lesion formation is a multistep process that includes not only endometrial tissue proliferation but also antiapoptosis and invasion, we investigated whether succinate acts as a chemoattractant for hESCs using a transwell migration assay. The average number of hESCs per field that migrated toward succinate after 48 h was significantly higher than that in the vehicle. This invasive effect was dose dependent and equivalent to that of CCL2 (Fig. 5A). Next, we analyzed the migration of hESCs into the wounded cell-free areas using a scratch assay. Time-lapse imaging revealed the ability of succinate to promote wound healing in hESCs in a concentration-dependent manner, reaching statistical significance upon treatment with 1 mM of succinate relative to the vehicle, reaching the maximal effect at the optimal concentration (Fig. 5B). We performed cell viability assays on hESCs and HMrSV5 cells treated with succinate for 24 and 48 h. OD450 analysis showed a significantly higher survival capacity of hESCs treated with succinate than that of the control (Fig. 5C), similar to the effect observed in HMrSV5 cells (Fig. 5D). Finally, we performed Annexin V/propidium iodide staining of hESCs and HMrSV5 cells treated with vehicle or succinate for 48 h. We evaluated the percentages of apoptotic and live cells and observed no differences between the succinate and vehicle (Fig. 5E,F).

### Succinate-stimulated peritoneal mesothelial cells recruit macrophages and boost ectopic growth and implantation of ESCs

**Fig. 6:**
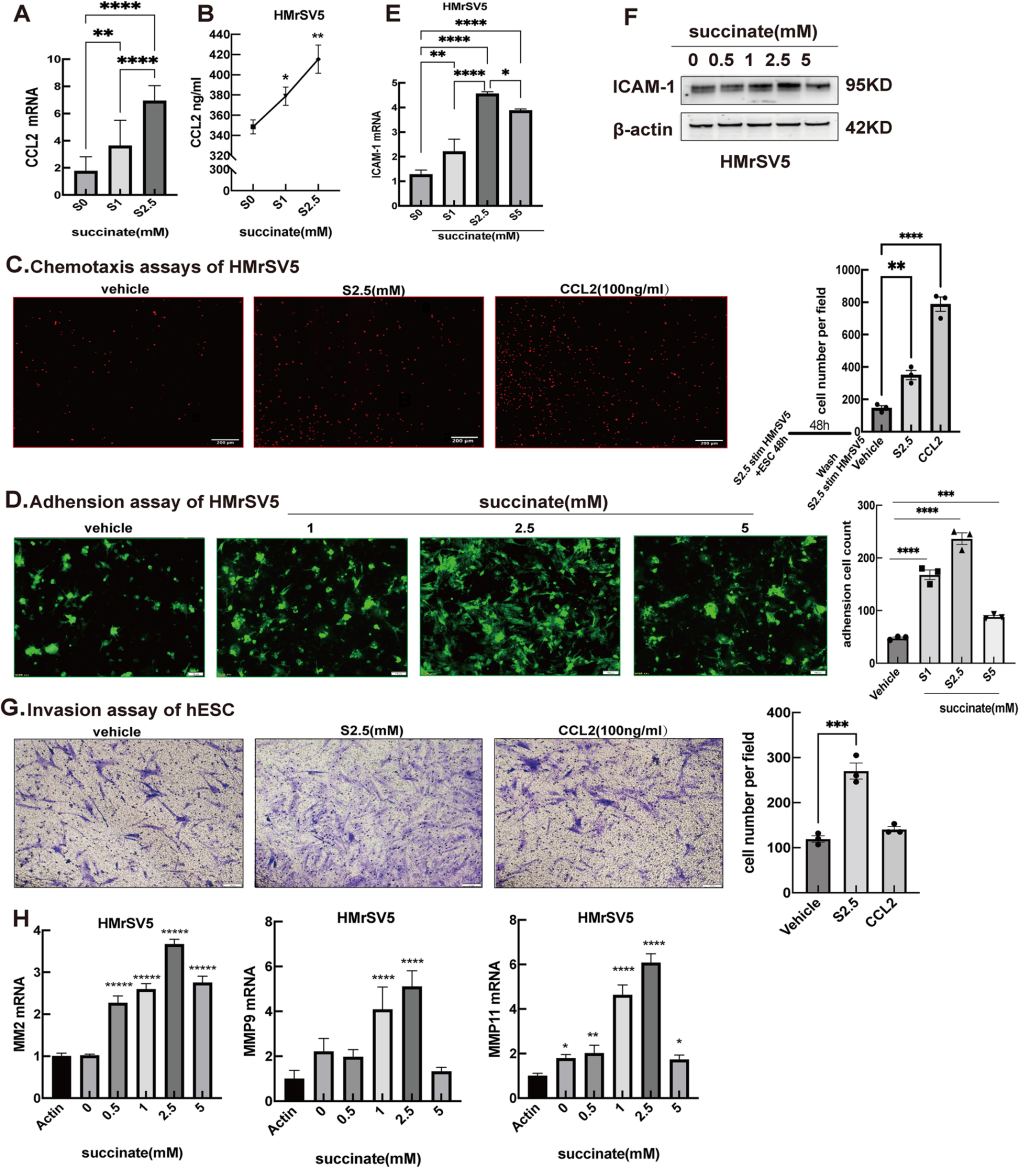
Succinate Enhances the Effect of Peritoneal mesothelial cells on Macrophage Recruitment and ESCs Adhesion. **A**, **B** RT-PCR was used to analyzed the CCL2 mRNA in HMrSV5 cells treated with vehicle or succinate (0, 1 and 2.5 mM) for 48 h, and ELISA was used to detected protein level of CCL2 (*n* = 3). All were analyzed by *one-way ANOVA* followed by *Dunnett’s* post hoc *test*, * *p* < 0.05, ** *p* < 0.01, *** *p* < 0.001, data are shown as mean ± SEM (CCL-2 group was positive control). **C** hESCs were seeded in upper chamber and HMrSV5 cells were treated with 2.5 mM succinate in lower chamber for 48 h, and then the hESCs was replaced with THP-1 cells for another 12 h. THP-1 cells were prelabeled with red fluorescent reagent CellTracker red. The chemotaxis assay for THP-1 cells was calculated as cell number per field (scale bar-200 μm) (red tracer staining THP-1 cell) (*n* = 3). *One-way ANOVA* followed by *Dunnett’s* post hoc *test*, * *p* < 0.05, ** *p* < 0.01, *** *p* < 0.001, data are shown as mean ± SEM (CCL-2 group was positive control). **D** Adhesion assays show hESCs adhere to HMrSV5 cells pretreated with different concentration of succinate for 48 h. hESCs(1 × 10^5^ cells/well) were prelabeled with green fluorescent reagent CellTracker green and seeded in the HMrSV5-well. Results are from 3 independent trials (n ≥ 3 for mimic) and data depicted as column mean graphs with error bars showing confidence intervals (scale bar-100um). **E** RT-PCR of ICAM-1 in HMrSV5 cells treated with vehicle or succinate (1,2.5 and 5 mM) for 48 h and analyzed by *one-way ANOVA* followed by *Dunnett’s* post hoc *test* (*n* = 3)*,* * *p* < 0.05, ** *p* < 0.01, *** *p* < 0.001, data are shown as mean ± SEM. **F** WB of ICAM-1 in HMrSV5 cells treated with different concentrations of succinate for 48 h. **G** Transwell migration assay of hESC in the presence of HMrSV5 cells with vehicle or 2.5 mM succinate treatment for 48 h. Media with CCL2 100 ng/ml. **H** RT-PCR of MMP2, MMP9 and MMP11 in HMrSV5 cells treated with succinate (0, 1, 2.5 and 5 mM) for 48 h and analyzed by *one-way ANOVA* followed by *Dunnett’s* post hoc *test* (*n* = 3), * *p* < 0.05, ** *p* < 0.01, *** *p* < 0.001, data are shown as mean ± SEM

Based on the above assays, we demonstrated that succinate promoted various endometriotic processes in hESCs, including cell survival, migration, adhesion and invasion. Because macrophages are the principal mediators of pathological EMs, we investigated whether succinate could recruit macrophages and promote the adhesion of hESCs to HMrSV5 cells. As we previously reported that endometrial stromal cells from the ectopic milieu continuously recruit monocytes and are beneficial for the expansion of monocyte-derived CCR2^+^ macrophages [26–28], we tested the ability of HMrSV5 cells to attract macrophages after succinate stimulation. Our results revealed that the treatment of HMrSV5 cells with succinate (0, 1, and 2.5 mM) for 48 h remarkably induced CCL2 gene expression and protein secretion (Fig. 6A,B). Next, in a co-culture system, where hESCs were placed in the upper chamber and HMrSV5 cells were exposed to 2.5 mM succinate in the lower chamber for 48 h, we subsequently replaced hESCs with THP-1 pre-labeled with CellTracker red; the co-culture was continued for an additional 12 h. The chemotaxis assay showed that HMrSV5 cells exposed to succinate readily attracted THP-1 cells (CCL2 treatment was used as the positive control) (Fig. 6C), suggesting that this chemotactic effect on monocytes/macrophages may be mediated by the CCL2, which is secreted by mesothelial cells after stress.

Although the theory of retrograde menstruation and immune disorders is helpful for understanding EMs, the mechanisms underlying the pathological factors and their roles in the ectopic implantation and aggressive growth of ESCs are still poorly understood. In the present study, we analyzed the effect of succinate on the adhesion of hESCs to PMCs. The adhesion assay showed that succinate triggered the adhesion of hESCs to HMrSV5 cells more strongly than the vehicle did, and that enhanced adhesion occurred when HMrSV5 cells were exposed to 2.5 mM succinate **(**Fig. 6D**)**. Further investigations showed that succinate-driven adhesion of hESCs to HMrSV5 cells might depend on the expression of ICAM-1 because succinate induced a concentration-dependent increase in ICAM-1 expression and of the adhesion capacity of HMrSV5 cells (Fig. 6E,F).

Therefore, succinate may play a pivotal role in the implantation growth of hESCs. Next, we investigated whether succinate derived from the ectopic milieu could induce hESC invasion and migration in the presence of HMrSV5 cells. This effect was evaluated using the Matrigel invasion assay. hESCs in the presence of HMrSV5 were stimulated with vehicle or 2.5 mM succinate for 48 h, and CCL2 treatment was used as a positive control. Indeed, we observed that 2.5 mM succinate strongly induced hESC invasion in the presence of HMrSV5 cells **(**Fig. 6G**)**. Matrix metalloproteinases (MMPs) are vital regulators of invasion and extracellular matrix remodeling. We also demonstrated that succinate (0.5–2.5 mM) stimulation induced the expression of MMPs,such as MMP2, MMP9 and MMP11, in HMrSV5 cells **(**Fig. 6H**)**.

**Fig. 7:**
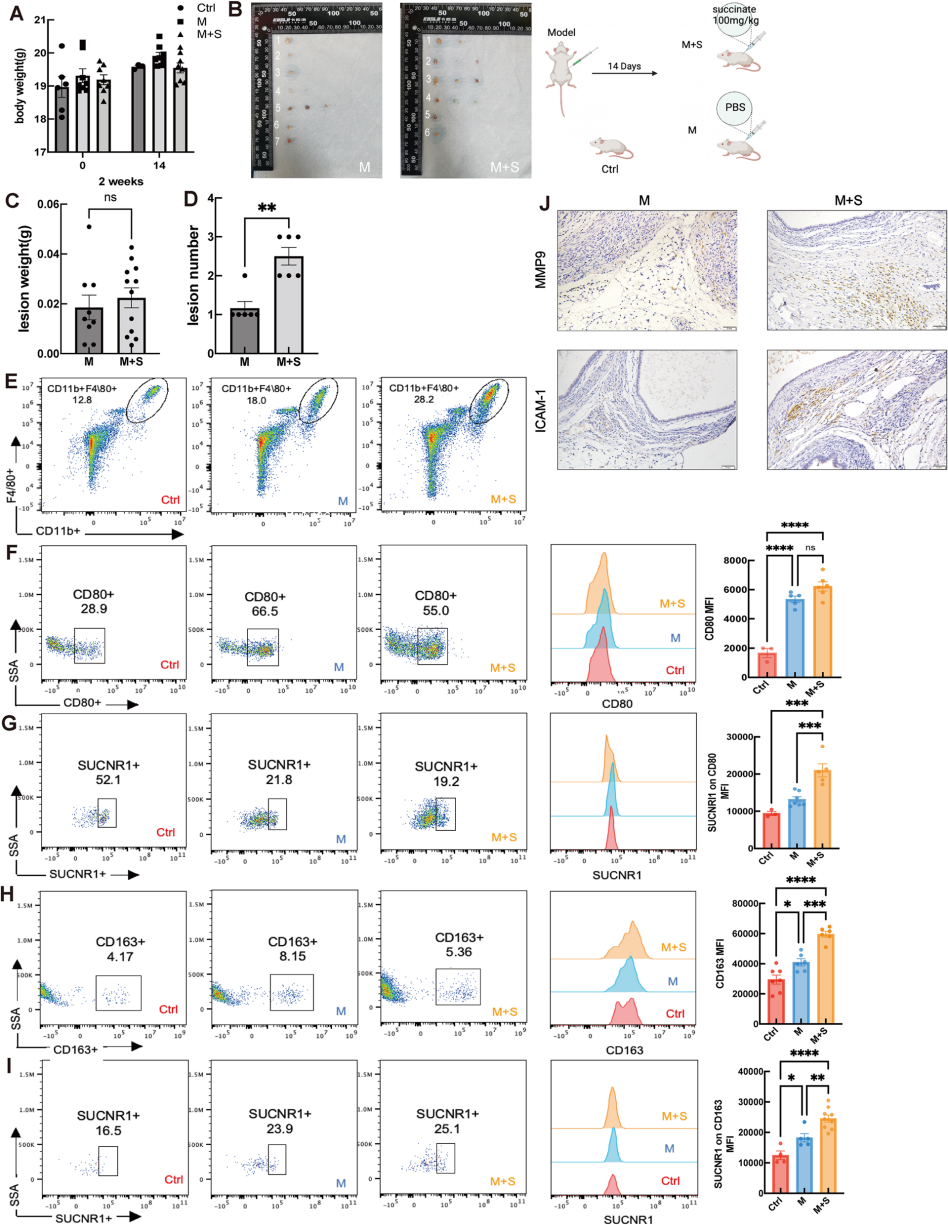
Succinate Induce the Enrichment of SUCNR1^+^ Macrophages and Ectopic Lesion Formation in vivo*.* A The wight change of mouse EMs model during the 2 weeks. Ctrl: PBS treatment; M: Model group; M + S: Model with succinate treatment group. The data are expressed as the mean ± SEM. (One-way ANOVA) *P < 0.05, **P < 0.01 and ***P < 0.001. B The macroscopic observation of the morphology of endometriosis-like lesions from endometriosis mouse models. M: Model group, M + S: Model with succinate treatment group. C-D The number and wight of EMs lesions was measured after the treatment of succinate (100 mg/kg) or PBS. M: Model group, M + S: Model with succinate treatment group. (*Student’s t-test*) *P < 0.05, **P < 0.01 and ***P < 0.001. E-I The levels of CD80, CD163 and SUCNR1 on peritoneal CD11b(+)F4/80(+) macrophages from mouse models were analyzed by using flow cytometry. MFI of SUCNR1 on M1 and M2 macrophages obtained were shown respectively. Ctrl: marked in red; M: marked in blue; M + S: marked in orange. (*One-way ANOVA*) *P < 0.05, **P < 0.01 and ***P < 0.001. J MMP9 and ICAM-1 expression in endometriosis-like lesion from model mice (M) and succinate exposing mice (M + S) by immunohistochemistry. M: Model group; M + S: Model with succinate treatment group. Original magnification: × 200

Further, in vivo analyses of the role of succinate in EMs progression were performed using an EMs allograft model. The body weight of the mice and the number and weight of lesions were recorded after treatment with either succinate (100 mg/kg) or PBS (Fig. 7A,B). Our results showed that the intraperitoneal injection of succinate did not affect the body weight of BALB/c mice (Fig. 7A) or ectopic lesion weight (Fig. 7C). Interestingly, the succinate-treated mice exhibited showed more ectopic lesions (Fig. 7D). To analyze the expression of SUCNR1 in M1 and M2 macrophages, SUCNR1 and CD80/CD163 on CD11b(+)F4/80(+) macrophages were assessed for mean fluorescence intensity using FCM. The results revealed that SUCNR1 expression was higher in both M1-like and M2-like peritoneal macrophages in the succinate-treated group than in the control and model groups (Fig. 7E-I). Similarly, succinate exposure increased the expression of ICAM-1 and MMP9 in ectopic lesions compared to that in the model group (Fig. 7J).

**Fig. 8:**
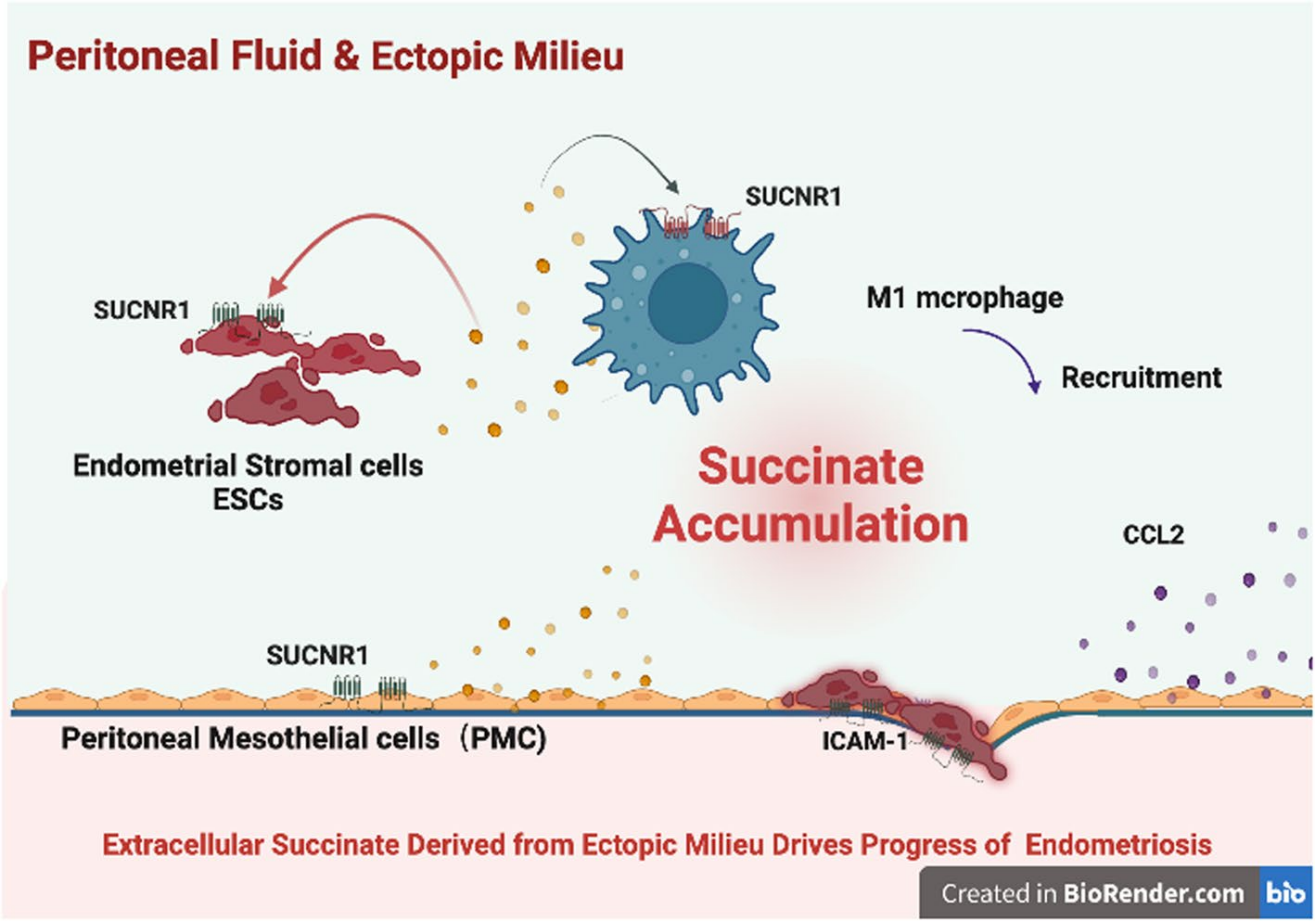
Schematic roles of Succinate from macrophage and peritoneal mesothelial cells in the progression of endometriosis by inducing inflammation and promoting ectopic growth. In the microenvironment of ectopic foci, exposure to inflammatory cytokines or contaction with ESCs obviously triggered succinate secretion from M1 polarized macrophages and PMCs, which are the main producers of succinate. Interestingly, compared with the normal endometrium, ESCs in ectopic tissues express high levels of SUCNR1. In PMCs, succinate promotes the auto-secretion of succinate and the expression of SUCNR1 in an autocrine amplification manner. In addition to promoting succinate accumulation, interactions between macrophages, mesothelial cells and ESCs in the ectopic milieu amplify the regulatory effect on cellular function via SUCNR1 signaling. Succinate promoted the survival, adhesion, invasion, and deep infiltration of ESCs via SUCNR1 signaling, leading to the formation of ectopic lesions in endometriosis. In conclusion, succinate in the ectopic milieu synergizes with polarized macrophages to exacerbate inflammation and facilitate endometriosis progress via succinate-SUCNR1-dependent mechanisms

In summary, these data indicate that succinate accumulation induced by polarized macrophages and PMCs enhances the aggressive implantation of ectopic ESCs and the adhesion between ESCs and PMCs, promoting the progression of EMs via SUCNR1 signaling Fig. 8 (created with BioRender.com).

## Discussion

Over the past few decades, metabolites have been considered as the vital players in metabolism have recently been proven to have key immune regulatory functions. Among these, succinate has notable multifaceted roles in the regulation of immune and metabolic functions [12, 20, 21, 29], dynamic changes, and selective cell release [30]. Notably, several pathogenic states such as obesity [17], diabetes [31], hypertension [32], and various inflammatory conditions [33] are associated with elevated levels of extracellular succinate in the body.

Our study identified an important axis comprising succinate and its cognate receptor SUCNR1 as potential drivers of ectopic endometrial survival and adhesion in the EMs milieu. As EMs progresses, succinate accumulates in the peritoneal fluid of EMs patients. Hypoactivity/deficiency or overproduction of SDH may cause succinate accumulation in the ectopic milieu, correlating with various clinical symptoms in EMs, such as dysmenorrhea. This association aligns with rAFS classification and the EFI.

In this study, we investigated the main sources of succinate in the extracellular fluid of patients with EMs. Upon stimulation or polarization with LPS, macrophages changed from oxidative phosphorylation to glycolysis, accompanied by elevated levels of intracellular and extracellular succinate [34–36]. Our data revealed that succinate was mainly derived from type 1 polarized macrophages. Surprisingly, PMCs produced high levels of succinate comparable to those produced by M1 macrophages. The conditions that trigger succinate secretion include stimulation by the inflammatory cytokine IL-6 and exposure of stromal cells. The elevation of succinate in the cell supernatant partly depended on the direct physical contact between mesothelial cells/macrophages, and hESCs. However, higher ability of succinate intake by M1 macrophages may also counteract the amount of succinate leaked into extracellular environment from ESC in co-culture system, which may partly explain the changes of succiante in the co-culture system of M1 and hESC. However, mechanistic insights into the effects of dysregulated succinate levels on ectopic cellular function in this pathology remain unavailable.

SUCNR1 is widely and heterogeneously expressed in various cell types throughout the body. The most well-studied SUCNR1-expressing cells are monocytes and macrophages [21, 34]. In addition, many non-immune tissues, including the intestine [10], placenta [11], skeletal muscle satellite [37], and endothelium [38], express SUCNR1 and respond to paracrine signaling in the form of succinate secretion in response to local pathologies. Inflammation and fibrosis associated with these pathologies may be, at least in part, attributable to chronic SUCNR1 agonism in tissue-resident cell populations. While the succinate-SUCNR1 interplay has been proposed as a molecular mechanism in rheumatoid arthritis [18] and intestinal inflammation [10], our data also imply a role for this pathway also in EMs formation. We confirmed the expression of SUCNR1 in ESCs and PMCs. FCM analysis highlighted differences in SUCNR1 expression among various stromal cells, which may be reflected in their responses to succinate stimuli via SUCNR1. The FCM and real-time PCR results demonstrated that SUCNR1 expression was higher in primary ectopic ESCs than in normal ESCs, suggesting that the succinate-SUCNR1 signal may be a potential driver of EMs. Indeed, we confirmed that succinate-SUCNR1 signaling is involved in the survival and adhesion of hESCs, influencing a battery of crucial steps in ectopic endometrial lesion formation. Functional experiments verified that succinate promoted hESC survival and invasion, as well as conferred antiapoptotic effects. Interestingly, exogenous succinate increased the mRNA and protein expression of SUCNR1 in HMrSV5 cells, further secreted proinflammatory CCL2 recruiting macrophages, and induced remarkable adhesion between HMrSV5 cells and hESCs, implying that these changes among cells in the ectopic milieu triggerred a vicious circle of EMs progression.

In our study, succinate induced macrophages to polarize toward the M1 phenotype, which is consistent with a previous study [34]. The assessment of IL-8 via ELISA, along with the assessment of the SUCNR1 membrane receptor and macrophage surface markers (CD80, CD86, CD163, and CD206) using FCM, provided consistent evidence supporting the proinflammatory potential of succinate stimulation. This response was comparable to that observed in LPS-stimulated macrophages. However, at a certain range, succinate endows macrophages with proinflammatory functions, whereas excessively high concentrations of succinate (5 mM) impair IL-8 production. Furthermore, extracellular succinate synergizes with LPS during macrophage polarization toward the M1 phenotype and proinflammatory functional transformation. Besides, it’s important to note that succinate is not specific to endometriosis and can be produced in various physiological and pathological conditions. Research has shown that pelvic inflammatory disease (PID) can also result in alterations in the metabolic profile of affected tissues, including changes in metabolites such as succinate [39]. Inflammatory processes can lead to an increase in succinate levels due to the activation of immune cells and the release of succinate as a byproduct of cellular metabolism. In the context of endometriosis, our findings highlight the importance of succinate-SUCNR1 signaling in macrophage polarization and suggest its role in immune regulation.

## Conclusion

Our results showed that EMs patients present high levels of succinate in the peritoneal fluid and increased SUCNR1 expression in ESCs. This succinate-SUCNR1 axis exacerbates the inflammatory activity of macrophages and ESC activation and plays a role in endometrial lesion formation and peritoneal adhesion. This is the first study to demonstrate the role of succinate and its receptors in EMs. We propose that, in the ectopic milieu of EMs patients, SUCNR1 signaling exacerbates inflammation and benefits the invasion, survival, and adhesive growth of ectopic ESCs, indicating a possible target for EMs treatment.

## Statistics

Spearman’s correlation analysis was used to analyze the correlation between extracellular succinate levels and clinical symptoms in humans. The diagnostic performance of succinate was determined using the area under the receiver operating characteristic curve (AUROC) analysis to assess the overall discriminatory power of these assays in predicting EMs progression. The continuous variables are expressed as mean ± SEM. Data from two groups were analyzed using Student’s t-test, whereas data from multiple groups were analyzed via one-way ANOVA using Tukey’s post-hoc test. Statistical analyses were performed using Statistical Package for the Social Sciences (SPSS Inc., Chicago, 26.0 version) and Prism5.0 software (GraphPad Software Inc.). Statistical significance was set at *P*-value < 0.05.

### Supplementary Information

**Additional file 1: Figure 1.** Succinate and SDHB expression in EMs milieu. (A) Succinate accumulation in PF of patients with EMs was confirmed by ELISA. (B) SDHB expression in normal endometrium (*n* = 5), and ectopic lesion (*n* = 3) by immunohistochemistry. Non-EMs: endometrium from patients without endometrioss; EMs: ectopic lesion from women with endometriosis. Original magnification: × 200. SDHB expression were detected in hESC line, primary normal ESC, and primary ectopic ESC using via western blot. hESC.L: hESC line; hESC.N: primary normal ESC; hESC.D: primary ectopic ESC. SDHB expression were detected in hESC line, primary normal ESC, and primary ectopic ESC using via real time PCR (One-way ANOVA, *P < 0.05). **Figure 2.** Succinate Amplified the polarization of M1 phenotype. B, D-E) Under the initial stimulus condition, relative mRNA expression levels of M1 markers (CD80, CD86), M2 markers (CD206, CD163) in both vehicle and succinate (0, 1, 2, 2.5 and 5 mM) group. (C) CD 86 expression were assay via FCM in THP-1 cells stimulated with single succinate or LPS, as well as a combination of both for 24 h. Points or bars in graphs represent mean ± SEM. Significant differences in relation to the vehicle group are shown by *P < 0.05, **P < 0.01, ***P < 0.001. (F-I) RT-PCR of IL-8, IL-1β, IL-6 in THP-1 derived macrophages treated with vehicle or succinate for 24 h. * *p* < 0.05, ** *p* < 0.01, *** *p* < 0.001, data are shown as mean ± SEM (*n* = 3). (J) SUCNR1 expression were assay via FCM in THP-1 cells stimulated with succinate(0, 0.5, 1, 2.5 and 5 mM) for 48 h. *one-way ANOVA* followed by *Dunnett’s* post hoc *test*, * *p* < 0.05, ** *p* < 0.01, *** *p* < 0.001, data are shown as mean ± SEM (*n* ≥ 3). **Figure 3.** Expression of Channel Proteins for succinate in EMs milieu. C) mRNA expression of transporters responsible for succinate exportation (MCT1) and succinate uptake (SLC26A6, SLC25A10) in hESC line, primary normal ESC, and primary ectopic ESC using via real time PCR. hESC.L: hESC line; hESC.N: primary normal ESC; hESC.D: primary ectopic ESC. (D-F) mRNA expression of transporters responsible for succinate exportation (MCT1) and succinate uptake (SLC26A6, SLC25A10) in hESC line, Student’s t test (t test) * *p* < 0.05, *** *p* < 0.001, **** *p* < 0.0001, data are shown as mean ± SEM (n ≥ 3). (G) MCT1 expression was detected in hESC line, primary normal ESC, and primary ectopic ESC using via western blot. (H) In the co-culture system, MCT1 expression was detected on hESC line, PMCs, and M1 macrophage using via FCM. *one-way* ANOVA followed by *Dunnett’s* post hoc *test*, ** *p* < 0.01, *** *p* < 0.001, **** *p* < 0.0001, data are shown as mean ± SEM (n ≥ 3).

## Data Availability

The metabolite data used and analysis during the current study are available from the corresponding author on reasonable request.
