# Supplementary material for: Extracellular succinate derived from ectopic milieu drives adhesion and implantation growth of ectopic endometrial stromal cells via the SUCNR1 signal in endometriosis
Source: Cell Commun Signal. 2024 Jan 30;22:82. doi: 10.1186/s12964-023-01415-7 (PMC10826047; doi:10.1186/s12964-023-01415-7)
Supplement: Supplementary file 2 — Additional file 1: Figure 1. Succinate and SDHB expression in EMs milieu. (A) Succinate accumulation in PF of patients with EMs was confirmed by ELISA. (B) SDHB expression in normal endometrium (n = 5), and ectopic lesion (n = 3) by immunohistochemistry. Non-EMs: endometrium from patients without endometrioss; EMs: ectopic lesion from women with endometriosis. Original magnification: × 200. SDHB expression were detected in hESC line, primary normal ESC, and primary ectopic ESC using via western blot. hESC.L: hESC line; hESC.N: primary normal ESC; hESC.D: primary ectopic ESC. SDHB expression were detected in hESC line, primary normal ESC, and primary ectopic ESC using via real time PCR (One-way ANOVA, *P < 0.05). Figure 2. Succinate Amplified the polarization of M1 phenotype. B, D-E) Under the initial stimulus condition, relative mRNA expression levels of M1 markers (CD80, CD86), M2 markers (CD206, CD163) in both vehicle and succinate (0, 1, 2, 2.5 and 5 mM) group. (C) CD 86 expression were assay via FCM in THP-1 cells stimulated with single succinate or LPS, as well as a combination of both for 24 h. Points or bars in graphs represent mean ± SEM. Significant differences in relation to the vehicle group are shown by *P < 0.05, **P < 0.01, ***P < 0.001. (F-I) RT-PCR of IL-8, IL-1β, IL-6 in THP-1 derived macrophages treated with vehicle or succinate for 24 h. * p < 0.05, ** p < 0.01, *** p < 0.001, data are shown as mean ± SEM (n = 3). (J) SUCNR1 expression were assay via FCM in THP-1 cells stimulated with succinate(0, 0.5, 1, 2.5 and 5 mM) for 48 h. one-way ANOVA followed by Dunnett’s post hoc test, * p < 0.05, ** p < 0.01, *** p < 0.001, data are shown as mean ± SEM (n ≥ 3). Figure 3. Expression of Channel Proteins for succinate in EMs milieu. C) mRNA expression of transporters responsible for succinate exportation (MCT1) and succinate uptake (SLC26A6, SLC25A10) in hESC line, primary normal ESC, and primary ectopic ESC using via real time PCR. hESC.L: hESC line; hESC [file 12964_2023_1415_MOESM1_ESM.zip › Supplementary/Supplementary Table 1.docx]

Supplementary Material

**Supplementary Table 1**

**The statistics of clinical parameters in patients with or without endometriosis**

| **Mean±SEM** | **Non-EMs(n=30)** | **EMs(n=36)** | ***p*** |
| --- | --- | --- | --- |
| **Age(y)** | 34.7±6.6 | 36.6±5.8 | 0.2600 |
| **Menstrual cycle**  Follicula phase:  Luteal phase: | 15(50)  15(50) | 18(50)  18(50) |  |
| **VAS scores** | 1.42±0.84 | 4.41±3.48 | 0.0020* |
| **Succinate (ng/ml) in peritoneal fluid** | 244.27±43.76 | 328.65±105.4 | 0.0001 |
| **rAFS**^a^ **stage**  I-II  III-IV | /  / | 12  24 |  |
| **rAFS scores:** | / | 78.59±32.58 |  |
| **EFI**^b^ **scores** | / | 6.22±1.85 |  |

^a^- revised American Fertility Society (rAFS, 1985); ^b^- endometriosis fertility index，EFI
